# Ten-year trends in major lifestyle risk factors using an ongoing population surveillance system in Australia

**DOI:** 10.1186/s12963-014-0031-z

**Published:** 2014-10-30

**Authors:** Anne W Taylor, Eleonora Dal Grande, Jing Wu, Zumin Shi, Stefano Campostrini

**Affiliations:** Population Research & Outcome Studies, Discipline of Medicine, The University of Adelaide, Adelaide, South Australia; Ca’ Foscari University, Venice, Italy; Department of Economics, Ca’ Foscari University, Venice, Italy

**Keywords:** Risk factors, Surveillance, Australia, Trends

## Abstract

**Background:**

Understanding how risk factors (tobacco, alcohol, physical inactivity, unhealthy diet, high blood pressure, and high cholesterol) change over time is a critical aim of public health. The associations across the social gradient over time are important considerations. Risk factor surveillance systems have a part to play in understanding the epidemiological distribution of the risk factors so as to improve preventive measures and design public health interventions for reducing the burden of disease.

**Methods:**

Representative, cross-sectional data were collected in South Australia using telephone interviews, conducted on a minimum of 600 randomly selected people (of all ages) each month. Data were collected from January 2004 to December 2013. Unadjusted prevalence over time, the relative percentage change over the 10 years, and the absolute change of the risk factors with sex, age group, and socio-economic status (SES) estimates are presented.

**Results:**

In total 55,548 adults (≥18 years) were interviewed (mean age = 47.8 years, 48.8% male). Decreases were apparent for insufficient physical activity, inadequate fruit and vegetables, smoking, and soft drink consumption of ≥500 ml/day. Increases were found over the 10 years for obesity, high cholesterol, diabetes, and for those with no risk factors. Apparent differences were noticeable by different sex, age, and SES categories. While increases in physical activity and fruit and vegetable consumption and decreases in smoking prevalence and multiple risk factors are to be expected in 2020–2021, the prevalence of obesity, high blood pressure, high cholesterol, and diabetes are expected to increase.

**Conclusions:**

Public health efforts in increasing the proportion of the population undertaking appropriate risk factor behavior are showing signs of success, with data from 2004 to 2013 showing encouraging trends. Deriving comparable trends over time by key demographics and SES variables provides evidence for policymakers and health planners to encourage interventions aimed at preventing chronic disease.

**Electronic supplementary material:**

The online version of this article (doi:10.1186/s12963-014-0031-z) contains supplementary material, which is available to authorized users.

## Background

Recent worldwide burden of disease studies highlight the epidemic of non-communicable diseases (NCD) and the important role played by major risk factors [1–3]. Most of the burden is attributed to just a few risk factors [3–5]. Understanding how the most cited behavioral-related factors (tobacco use, the harmful use of alcohol, physical inactivity, an unhealthy diet) and other risk factors such as high blood pressure and high cholesterol change over time, particularly in response to public health and health promotion action, is a critical aim of public health [5]. In addition, the associations across the social gradient over time are an important consideration [5–7] as masked behind many broad estimates are changes in trends by demographic, social, and economic indicators [2].

The behavioral-related risk factors have been shown to be independently associated with cardiovascular disease (CVD), type 2 diabetes, and other chronic diseases as well as increased health service costs and premature morbidity [8,9]. Health promoting campaigns, policy adaptions, and government interventions aimed at modification of risk factors are common [4,10–13].

An important way to gain a better understanding of these risk factors is demonstrated by monitoring their prevalence through risk factor surveillance systems, which have been established in many parts of the world [14–18]. These surveillance systems are essential for better understanding of the epidemiological distribution of risk factors to improve preventive measures and design public health interventions for reducing NCDs and the social and economic burden they represent. They also provide a better understanding of population strengths, highlight vulnerable populations, and detail changes in populations and across regions. Ongoing risk factor surveillance systems are also able to monitor the impact of policy planning, implementation, and evaluation over time. As argued by Hallal [19], notable gaps remain internationally in providing evidence and subsequent development of policies and programs to increase/decrease prevalence of risk factors and to reduce the burden of NCDs. The absence of continuous surveillance systems implemented at the national level is a major gap in preventing many countries from analyzing trend data [1,2,19].

This analysis provides a descriptive analysis of cross-sectional trends of established risk factors over a 10-year period. Recently, the consumption of soft drinks has become prominent as a risk factor for ill health [20], and this is also included in the following analyses. As diabetes is a major risk factor for CVD, prevalence estimates for self-reported diabetes status are also included, as is a derived variable assessing multiple risk factors.

## Methods

The South Australian Monitoring and Surveillance System (SAMSS) is a telephone monitoring system designed to systematically monitor chronic disease, risk factors, and other health-related issues on a regular and ongoing basis [21]. Commencing in July 2002, a representative cross-sectional sample is randomly selected each month from all households in South Australia (SA) with a telephone connected and the number listed in the telephone directory. A letter of introduction is sent to the selected household and the person who was last to have a birthday within a 12-month period is chosen for interview. Surrogate interviews are taken with a responsible adult for selected respondents under the age of 16 years.

A trained interviewer, via a Computer-Assisted Telephone Interview (CATI) system, conducts the interviews. Data are collected by a contracted agency and interviews are conducted in English by trained health interviewers. Each interview takes approximately 15 minutes. At least 10 call-backs are made to the telephone number selected. Replacement interviews for persons who could not be contacted or interviewed are not permitted. Interviews are conducted on a minimum of 600 randomly selected people (of all ages) each month. Of each interviewer’s work, 10% is selected at random for validation by a supervisor. The current analysis used data collected in the period January 2004 to December 2013 for respondents aged 18 years and over with data for each two-year time period combined. The median response rate of SAMSS for this period was 64.9%.

All respondents gave informed consent to undertaking the interview. Ethics approval was obtained from the ethics committee of the Department of Health and Ageing, SA.

Regarding physical activity, respondents were asked to provide the time they spent undertaking walking, moderate, or vigorous physical activity over the past week. The time is summed, with the time spent undertaking vigorous activity multiplied by a factor of two to account for its greater intensity, in order to provide an indication as to whether respondents are undertaking a sufficient level of physical activity to provide a health benefit. This is defined as 150 minutes or more of activity each week [22] and has been recoded into no activity, active but not sufficient, and sufficient activity [23].

Body mass index (BMI) is derived from self-reported weight and height and recoded into three categories (underweight/normal, overweight, and obese) [24]. Respondents were asked how many servings of fruit and how many servings of vegetables they eat each day with the recommendation being at least two servings of fruit and five servings of vegetables each day [25]. If respondents were not eating the recommended servings of fruit and vegetables they were classified as inadequate fruit and vegetable (consuming <5 vegetable servings and/or <2 fruit servings per day).

Questions relating to alcohol included the two questions recommended by the Australian National Health and Medical Research Council [26]. These are “how often do you drink alcohol” and “on a day when you drink alcohol how many drinks do you usually have”. Drinking more than two standard drinks on any day has been deemed to increase the lifetime risk of harm from alcohol-related disease or injury [26]. Smoking status (current, ex-, or nonsmoker) was also assessed. All of the aforementioned risk factor questions have been assessed for validity and reliability in the Australian CATI setting [27].

Respondents were asked if a doctor had ever told them they had diabetes and if a doctor had ever told them they have or they were currently receiving treatment or medication for high blood pressure or high cholesterol. Since 2008 all respondents were asked their soft drink consumption (mls per day) with ≥500 mls per day deemed risky. In addition, a multiple risk factor variable was created from the eight risk factors assessed continually (physical inactivity, lifetime risk of harm from alcohol-related disease or injury, current smoking, inadequate consumption of fruit and/or vegetables, current high blood pressure, current high cholesterol, diabetes, and obesity).

Demographic and socio-economic variables included in the analyses consisted of age, gender, and socio-economic status (SES) using postcode classified into the Socio-Economic Index For Areas (SEIFA) 2006 Index of Relative Socio-Economic Disadvantage quintiles [28].

The data were weighted by age, sex, and area of residence to reflect the structure of the population in SA to the latest Australian Bureau of Statistics Census or Estimated Residential Population data. Probability of selection in the household was calculated based on the number of people in the household and the number of listings in the telephone directory. Weighting is used to correct for disproportionality of the sample with respect to the population of interest. In total, approximately 60% of the total SA households are included in the telephone directory. The weighting of the data allows for most bias to be compensated as a result of the non-contacted households or for any gender or age-group discrepancies. The SAMSS questionnaire has been approved by the SA Health Ethics of Human Research Committee.

Data were analysed using SPSS for Windows Version 19.0 and Stata Version 12.0. Prevalence estimates were assessed for each risk factor and relative percentage (absolute) change calculated. Trends were tested using logistic regression. Each prevalence estimate was then age-adjusted and 2020–2021 projections calculated.

## Results

From January 2004 to December 2013, 55,548 adults (≥18 years) were interviewed (mean age = 47.8 years, 48.8% male). Table 1 highlights the demographic characteristics of respondents. Tables 2, 3, 4 and 5 detail the unadjusted prevalence over time, the relative percentage change over the 10 years, and the absolute change of the risk factors (insufficient physical activity, obesity, smoking, inadequate nutrition, adverse alcohol intake, adverse soft drink consumption, diabetes, current high blood pressure and current high cholesterol, and a variable highlighting those who had multiple risk factors). Sex, age group, and SES estimates are also presented for all risk factors.

**Table 1:** **Demographic characteristics of respondents aged 18 years and over, 2004 to 2013**

	**Year**				
	**2004-2005**	**2006-2007**	**2008-2009**	**2010-2011**	**2012-2013**
**Age** **, (years)**					
18 – 39	37.3	36.9	36.4	35.4	34.2
40 – 64	43.2	43.3	43.7	44.6	45.0
65 and over	19.5	19.7	19.9	20.1	20.8
**Sex** **(%)**					
Males	48.8	49.1	48.8	48.7	48.6
Females	51.2	50.9	51.2	51.3	51.4
**Area of residence** **(%)**					
Metropolitan Adelaide	73.1	73.0	72.9	72.6	72.3
Country SA	26.9	27.0	27.1	27.4	27.7
**Marital status** **(%)**					
Married/Living with partner	68.2	68.4	67.2	64.7	65.9
Separated/Divorced	6.9	6.9	6.6	6.9	6.6
Widowed	6.1	6.1	6.5	5.9	5.7
Never married	18.7	18.5	19.7	22.5	21.9
**Education** **(%)**					
No schooling to secondary	54.8	51.7	51.2	50.3	46.1
Trade, certificate, diploma	25.2	26.7	25.6	27.2	30.6
Degree or higher	20.0	21.7	23.2	22.5	23.3
**SEIFA** **(%)**					
Low/lowest quintile (most disadvantaged)	35.9	36.6	36.0	37.1	37.2
Middle quintile	20.4	20.0	20.5	20.5	21.1
High/highest quintile (most advantaged)	43.8	43.4	43.5	42.4	41.7
**Gross annual household income** **(%)**					
≤ $40,000	40.1	34.7	30.1	28.5	26.8
$40,001 to $80,000	34.6	33.7	32.0	30.7	37.8
≥ $80,001	25.3	31.6	37.9	40.8	35.4
**Home ownership status** **(%)**					
Own/being purchased	83.2	84.9	85.1	85.3	84.2
Rent or others	16.8	15.1	14.9	14.7	15.8

**Table 2:** **Unadjusted prevalence of insufficient physical activity and unhealthy diet and smoking by age, sex and socio-economic status, 2014 to 2013**

		**Year; prevalence, % (95% CI)**					**Relative change % (absolute change)**	***P*** **value for trend**
		**2004-2005**	**2006-2007**	**2008-2009**	**2010-2011**	**2012-2013**		
**Insufficient physical activity**								
*Overall*		61.7 (60.6-62.7)	59.6 (58.5-60.8)	59.4 (58.2-60.6)	59.7 (58.5-60.9)	59.6 (58.2-60.9)	-3.4 (-2.1)	0.005
Sex	Males	59.4 (57.8-61.0)	58.2 (56.5-60.0)	57.7 (55.9-59.5)	58.1 (56.2-59.9)	58.1 (55.9-60.2)	-2.2 (-1.3)	0.174
	Females	63.8 (62.4-65.2)	61.0 (59.5-62.4)	61.1 (59.5-62.6)	61.3 (59.7-62.8)	61.0 (59.3-62.7)	-4.4 (-2.8)	0.009
Age (y)	18 – 39	54.4 (52.3-56.5)	52.6 (50.3-54.9)	50.8 (48.2-53.3)	53.4 (50.9-55.9)	51.0 (47.9-54.1)	-6.3 (-3.4)	0.017
	40 – 64	63.2 (61.7-64.7)	60.2 (58.7-61.7)	60.5 (58.9-62.0)	60.0 (58.3-61.6)	61.1 (59.3-62.9)	-3.3 (-2.1)	0.046
	65 and over	72.2 (70.7-73.8)	71.6 (70.0-73.1)	73.0 (71.6-74.4)	70.4 (69.0-71.7)	70.3 (68.9-71.7)	-2.6 (-1.9)	0.097
SES	Low/lowest quintile	65.7 (64.0-67.4)	63.8 (62.0-65.6)	62.1 (60.1-64.0)	62.9 (61.0-64.9)	64.7 (62.5-67.0)	-1.5 (-1.0)	0.244
	Middle quintile	61.5 (59.1-63.8)	61.3 (58.9-63.7)	63.9 (61.4-66.4)	61.1 (58.5-63.8)	57.8 (55.7-61.6)	-6.0 (-3.7)	0.074
	High/highest quintile	58.6 (56.9-60.2)	55.3 (53.5-57.1)	55.1 (53.2-56.9)	56.3 (54.5-58.1)	55.5 (53.4-57.6)	-5.3 (-3.1)	0.022
**Daily vegetable (<5 servings) and/or fruit (<2 servings) intake**								
*Overall*		54.5 (53.4-55.5)	54.1 (53.0-55.2)	49.8 (48.6-50.9)	51.2 (50.0-52.4)	51.5 (50.1-52.8)	-5.5 (-3.0)	<0.001
Sex	Males	60.9 (59.4-62.5)	61.3 (59.6-62.9)	55.3 (57.1-46.0)	58.0 (56.2-59.8)	60.0 (57.9-62.0)	-1.5 (-0.9)	0.015
	Females	48.3 (46.9-49.7)	47.2 (45.8-48.7)	44.5 (50.9-55.9)	44.7 (43.2-46.2)	43.5 (41.7-45.1)	-9.9 (-4.8)	<0.001
Age (y)	18 – 39	60.9 (58.9-62.9)	58.8 (56.5-61.0)	53.4 (48.7-51.8)	54.6 (52.2-57.1)	53.0 (49.9-56.1)	-13.0 (-7.9)	<0.001
	40 – 64	52.8 (51.3-54.3)	53.2 (51.7-54.8)	50.3 (40.4-43.5)	51.3 (49.7-53.0)	53.0 (51.2-54.8)	0.4 (0.2)	0.571
	65 and over	45.9 (44.1-47.6)	47.3 (45.6-49.0)	41.9 (40.4-43.5)	44.6 (43.2-46.1)	45.6 (44.1-47.0)	-0.7 (-0.3)	0.352
SES	Low/lowest quintile	57.2 (55.4-58.9)	57.1 (55.3-59.0)	52.6 (50.7-54.6)	55.2 (53.2-57.1)	55.8 (53.6-58.1)	-2.4 (-1.4)	0.066
	Middle quintile	54.9 (52.6-57.2)	53.3 (50.9-55.7)	51.5 (49.0-54.1)	52.2 (49.5-54.8)	53.6 (50.1-56.5)	-2.4 (-1.3)	0.263
	High/highest quintile	52.1 (50.4-53.7)	52.0 (50.2-53.7)	46.5 (44.6-48.3)	47.1 (45.3-49.0)	46.5 (44.4-48.6)	-10.7 (-5.6)	<0.001
**Smoking**								
*Overall*		19.4 (18.5-20.3)	17.8 (16.9-18.7)	15.5 (14.6-16.4)	15.4 (14.5-16.3)	13.4 (12.4-14.3)	-30.9 (-6.0)	<0.001
Sex	Males	21.5 (20.1-22.8)	20.8 (19.3-22.3)	17.5 (16.1-18.9)	17.7 (16.2-19.2)	16.2 (14.5-17.8)	-24.7 (-5.3)	<0.001
	Females	17.4 (16.2-18.5)	14.9 (13.8-16.0)	13.6 (12.5-14.6)	13.2 (12.1-14.3)	10.7 (9.6-11.8)	-38.5 (-6.7)	<0.001
Age (y)	18 – 39	25.3 (23.4-27.2)	23.5 (21.5-25.5)	19.2 (17.3-21.2)	17.9 (16.0-19.9)	14.6 (12.5-16.7)	-42.3 (-10.7)	<0.001
	40 – 64	19.7 (18.5-20.9)	18.0 (16.8-19.2)	16.5 (15.4-17.7)	17.9 (16.7-19.2)	15.8 (14.5-17.2)	-19.8 (-3.9)	<0.001
	65 and over	7.2 (6.3-8.1)	6.6 (5.7-7.4)	6.3 (5.6-7.1)	5.2 (4.6-5.8)	5.9 (5.3-6.6)	-18.1 (-1.3)	0.018
SES	Low/lowest quintile	23.2 (21.6-24.8)	21.7 (20.1-23.3)	20.3 (18.7-22.0)	19.0 (17.4-20.7)	15.7 (14.1-17.3)	-32.3 (-7.5)	<0.001
	Middle quintile	19.4 (17.5-21.4)	18.2 (16.1-20.3)	14.8 (13.0-16.7)	15.1 (13.1-17.1)	16.1 (13.6-18.6)	-17.0 (-3.3)	<0.001
	High/highest quintile	16.2 (14.9-17.5)	14.3 (12.9-15.6)	11.7 (10.6-12.9)	12.3 (11.1-13.6)	9.9 (8.6-11.2)	-38.9 (-6.3)	<0.001

**Table 3:** **Unadjusted prevalence of long term risk of harm from alcohol, soft drink consumption, and obesity by age, sex, and socio-economic status, 2004 to 2013**

		**Year; prevalence, % (95% CI)**					**Relative change % (absolute change)**	***P*** **value for trend**
		**2004-2005**	**2006-2007**	**2008-2009**	**2010-2011**	**2012-2013**		
**Long-term risk of harm from alcohol**								
*Overall*		3.9 (3.5-4.3)	3.8 (3.4-4.3)	4.1 (3.6-4.6)	3.8 (3.3-4.2)	3.9 (3.4-4.4)	0 (0)	0.833
Sex	Males	3.9 (3.3-4.5)	4.4 (3.8-5.1)	4.6 (3.9-5.4)	4.2 (3.5-4.9)	4.2 (3.4-5.0)	7.7 (0.3)	0.691
	Females	4.0 (3.3-4.5)	3.2 (2.7-3.8)	3.6 (3.0-4.2)	3.4 (2.9-4.0)	3.6 (3.0-4.2)	-10.0 (-0.4)	0.473
Age (y)	18 – 39	4.4 (3.6-5.3)	4.3 (3.3-5.1)	4.6 (3.6-5.7)	4.0 (3.0-5.0)	3.4 (2.4-4.5)	-22.7 (-1.0)	0.033
	40 – 64	4.3 (3.7-4.9)	4.2 (3.6-4.8)	4.6 (3.9-5.2)	4.4 (3.8-5.0)	5.0 (4.3-5.8)	16.3 (0.7)	0.074
	65 and over	2.2 (1.7-2.7)	2.2 (1.7-2.7)	2.2 (1.8-2.7)	2.1 (1.7-2.5)	2.1 (1.7-2.5)	-4.5 (-0.1)	0.768
SES	Low/lowest quintile	3.7 (3.1-4.4)	3.9 (3.2-4.6)	4.0 (3.2-4.8)	4.2 (3.4-5.1)	3.7 (2.9-4.5)	-2.6 (-0.1)	0.835
	Middle quintile	4.2 (3.2-5.1)	3.5 (2.6-4.4)	3.9 (2.9-4.9)	3.9 (3.0-4.8)	4.1 (2.8-5.4)	-2.4 (-0.1)	0.848
	High/highest quintile	3.9 (3.3-4.6)	3.9 (3.2-4.7)	4.3 (3.5-5.0)	3.3 (2.7-3.9)	3.9 (3.2-4.6)	0 (0)	0.454
**Soft drink consumption (≥500 ml/day)**								
*Overall*				11.8 (10.9-12.7)	9.4 (8.6-10.1)	7.7 (6.8-8.5)	-34.7 (-4.1)	<0.001
Sex	Males			15.9 (14.3-17.4)	12.4 (11.2-13.7)	10.3 (8.7-11.9)	-35.2 (-5.6)	<0.001
	Females			7.8 (6.9-8.8)	6.4 (5.6-7.3)	5.2 (4.4-6.0)	-33.3 (-2.6)	<0.001
Age (y)	18 – 39			17.9 (15.8-20.1)	13.1 (11.4-14.8)	10.1 (8.0-12.1)	-43.6 (-7.8)	<0.001
	40 – 64			10.1 (9.1-11.1)	9.1 (8.1-10.1)	8.0 (6.9-9.1)	-20.8 (-2.1)	<0.001
	65 and over			4.0 (3.3-4.7)	3.3 (2.8-3.9)	3.3 (2.5-3.5)	-17.5 (-0.7)	0.062
SES	Low/lowest quintile			15.7 (14.0-17.5)	12.2 (10.7-13.6)	10.0 (8.4-11.7)	-36.3 (-5.7)	<0.001
	Middle quintile			11.0 (8.9-13.2)	9.0 (7.4-10.7)	8.1 (6.0-10.2)	-26.4 (-2.9)	0.001
	High/highest quintile			8.8 (7.6-10.0)	7.1 (6.1-8.0)	5.3 (4.3-6.3)	-39.8 (-3.5)	<0.001
**Obesity**								
*Overall*		17.3 (16.6-18.1)	19.3 (18.4-20.1)	20.0 (19.1-20.9)	22.5 (21.5-23.5)	21.9 (20.8-23.0)	26.6 (4.6)	<0.001
Sex	Males	17.1 (16.0-18.3)	19.4 (18.1-20.8)	19.9 (18.5-21.3)	22.0 (20.5-23.6)	21.9 (20.1-23.7)	28.1 (4.8)	<0.001
	Females	17.6 (16.5-18.6)	19.1 (18.0-20.2)	20.1 (18.9-21.2)	22.9 (21.6-24.2)	21.9 (20.5-23.3)	24.4 (4.3)	<0.001
Age (y)	18 – 39	12.6 (11.3-14.0)	14.3 (12.7-15.9)	15.4 (13.4-17.2)	16.6 (14.7-18.5)	15.8 (13.4-18.3)	25.4 (3.2)	<0.001
	40 – 64	22.2 (20.9-23.4)	24.9 (23.5-26.2)	25.0 (23.7-26.3)	28.3 (26.7-29.8)	27.0 (25.4-28.6)	21.6 (4.8)	<0.001
	65 and over	15.6 (14.3-16.9)	16.2 (14.9-17.5)	17.4 (16.3-18.6)	20.1 (18.9-21.3)	20.8 (19.7-22.0)	33.3 (5.2)	<0.001
SES	Low/lowest quintile	21.6 (20.1-23.0)	22.9 (21.4-24.5)	25.0 (23.4-26.7)	27.0 (25.2-28.8)	27.4 (25.4-29.5)	26.9 (5.8)	<0.001
	Middle quintile	16.9 (15.2-18.6)	18.6 (16.8-20.4)	20.6 (18.6-22.6)	22.6 (20.4-24.8)	20.4 (18.2-22.7)	20.7 (3.5)	<0.001
	High/highest quintile	14.0 (12.9-15.2)	16.5 (15.2-17.8)	15.5 (14.3-16.7)	18.5 (17.1-19.9)	17.6 (16.0-19.1)	25.7 (3.6)	<0.001

**Table 4:** **Unadjusted prevalence of high blood pressure, high cholesterol, and diabetes by age, sex, and socio-economic status, 2004 to 2013**

		**Year; prevalence, % (95% CI)**					**Relative change % (absolute change)**	***P*** **value for trend**
		**2004-2005**	**2006-2007**	**2008-2009**	**2010-2011**	**2012-2013**		
**High blood pressure**								
*Overall*		18.9 (18.2-19.6)	19.0 (18.2-19.7)	19.7 (18.9-20.4)	21.2 (20.4-22.0)	21.1 (20.3-22.0)	11.6 (2.2)	<0.001
Sex	Males	18.2 (17.1-19.3)	17.9 (16.8-18.9)	19.2 (18.1-20.3)	20.3 (19.0-21.5)	20.4 (19.0-21.7)	12.1 (2.2)	<0.001
	Females	19.6 (18.6-20.5)	20.0 (19.1-21.0)	20.1 (19.2-21.1)	22.0 (21.0-23.0)	21.8 (20.8-22.9)	11.2 (2.2)	0.001
Age (y)	18 – 39	2.2 (1.5-2.8)	2.2 (1.6-2.8)	1.9 (1.2-2.5)	3.1 (2.3-4.0)	1.7 (0.9-2.5)	-22.7 (-0.5)	0.941
	40 – 64	18.7 (17.6-19.9)	18.5 (17.4-19.6)	20.1 (18.9-21.2)	20.7 (19.5-22.0)	20.8 (19.5-22.2)	11.2 (2.1)	<0.001
	65 and over	51.2 (49.4-52.9)	51.3 (49.6-53.0)	51.5 (49.9-53.1)	53.9 (52.5-55.4)	53.6 (52.2-55.1)	4.7 (2.4)	0.024
SES	Low/lowest quintile	20.3 (19.1-21.5)	21.1 (19.9-22.4)	21.4 (20.1-22.7)	24.6 (23.1-26.0)	22.6 (21.1-24.1)	11.3 (2.3)	<0.001
	Middle quintile	19.8 (18.2-21.4)	21.5 (19.8-23.2)	20.8 (19.1-22.5)	20.6 (18.8-22.3)	21.9 (20.0-23.8)	10.6 (2.1)	0.235
	High/highest quintile	17.3 (16.2-18.4)	16.0 (15.0-17.1)	17.7 (16.6-18.8)	18.5 (17.3-19.6)	19.5 (18.2-20.8)	12.7 (2.2)	<0.001
**High blood cholesterol**								
*Overall*		14.6 (13.9-15.2)	14.6 (13.9-15.2)	15.9 (15.2-16.5)	16.9 (16.1-17.6)	17.7 (16.8-18.6)	21.2 (3.1)	<0.001
Sex	Males	15.1 (14.0-16.1)	14.6 (13.6-15.6)	16.1 (15.0-17.2)	17.4 (16.2-18.7)	18.0 (16.5-19.4)	19.2 (2.9)	<0.001
	Females	14.1 (13.3-14.9)	14.6 (13.7-15.4)	15.6 (14.7-16.5)	16.3 (15.4-17.2)	17.4 (16.4-18.4)	23.4 (3.3)	<0.001
Age (y)	18 – 39	2.4 (1.7-3.1)	2.1 (1.5-2.7)	2.4 (1.6-3.2)	2.1 (1.4-2.8)	2.5 (1.0-4.0)	4.2 (0.1)	0.860
	40 – 64	16.2 (15.2-17.3)	15.8 (14.8-16.9)	17.6 (16.5-18.7)	19.0 (17.7-20.3)	18.8 (17.5-20.1)	16.0 (2.6)	<0.001
	65 and over	34.2 (32.5-35.8)	35.1 (33.5-36.8)	36.6 (35.1-38.2)	38.1 (36.7-39.6)	40.3 (33.8-41.7)	17.8 (6.1)	<0.001
SES	Low/lowest quintile	15.2 (14.1-16.3)	15.9 (14.8-17.1)	17.4 (16.2-18.6)	18.8 (17.5-20.1)	19.5 (17.9-21.2)	28.3 (4.3)	<0.001
	Middle quintile	14.9 (13.4-16.3)	15.2 (13.7-16.6)	16.0 (14.5-17.4)	16.7 (15.1-18.4)	17.2 (15.5-18.9)	15.4 (2.3)	0.011
	High/highest quintile	13.9 (13.0-14.9)	13.1 (12.1-14.1)	14.5 (13.5-15.5)	15.2 (14.1-16.3)	16.3 (15.1-17.6)	17.3 (2.4)	<0.001
**Diabetes**								
*Overall*		6.8 (6.4-7.3)	7.2 (6.7-7.7)	7.6 (7.0-8.1)	7.8 (7.3-8.4)	8.1 (7.6-8.7)	19.1 (1.3)	<0.001
Sex	Males	6.4 (5.7-7.0)	7.1 (6.4-7.8)	8.0 (7.2-8.8)	8.2 (7.4-9.0)	7.8 (7.1-8.6)	21.9 (1.4)	<0.001
	Females	7.2 (6.6-7.9)	7.3 (6.56-8.0)	7.2 (6.5-7.8)	7.5 (6.8-8.2)	8.4 (7.6-9.2)	16.7 (1.2)	0.021
Age (y)	18 – 39	1.9 (1.4-2.4)	2.2 (1.6-2.8)	2.8 (1.9-3.7)	2.3 (1.5-3.0)	2.3 (1.4-3.1)	21.1 (0.4)	0.262
	40 – 64	7.2 (6.4-7.9)	7.0 (6.2-7.7)	7.0 (6.3-7.7)	8.1 (7.2-9.0)	8.0 (7.1-8.8)	11.1 (0.8)	0.019
	65 and over	15.5 (14.2-16.8)	17.0 (15.7-18.3)	17.6 (16.4-18.7)	17.1 (15.9-18.2)	18.1 (17.0-19.3)	16.8 (2.6)	0.037
SES	Low/lowest quintile	8.2 (7.3-9.0)	9.1 (8.2-10.0)	8.8 (8.0-9.7)	10.0 (9.0-10.9)	9.4 (8.4-10.3)	16.0 (1.3)	0.019
	Middle quintile	7.5 (6.4-8.7)	6.6 (5.6-7.5)	7.7 (6.5-9.1)	8.2 (7.0-9.5)	8.3 (7.2-9.5)	10.7 (0.8)	0.069
	High/highest quintile	5.4 (4.8-6.1)	5.9 (5.2-6.6)	6.4 (5.7-7.2)	5.8 (5.1-6.5)	7.0 (6.1-7.8)	19.6 (1.6)	0.006

**Table 5:** Unadjusted prevalence of multiple risk factors* by age, sex and socio-economic status, 2004 to 2013

		**Year; prevalence, % (95% CI)**					**Relative change % (absolute change)**	***P*** **value for trend**
		**2004-2005**	**2006-2007**	**2008-2009**	**2010-2011**	**2012-2013**		
**No risk factors (0/8)**								
*Overall*		11.3 (10.6-12.0)	13.1 (12.3-13.9)	13.6 (12.8-14.6)	13.2 (12.4-14.1)	14.0 (13.0-15.1)	23.9 (2.7)	<0.001
Sex	Males	10.3 (9.3-11.4)	11.4 (10.3-12.6)	12.1 (10.9-13.5)	11.4 (10.3-12.7)	11.3 (10.0-12.7)	9.7 (1.0)	0.287
	Females	12.2 (11.3-13.3)	14.8 (13.7-15.9)	15.1 (13.9-16.4)	15.0 (13.8-16.3)	16.7 (15.3-18.3)	36.9 (4.5)	<0.001
Age (y)	18 – 39	15.0 (13.5-16.5)	16.6 (15.0-18.4)	19.5 (17.5-21.6)	18.3 (16.5-20.3)	20.6 (18.3-23.1)	37.3 (5.6)	<0.001
	40 – 64	10.8 (9.8-11.8)	13.1 (12.1-14.2)	11.9 (11.0-13.0)	12.5 (11.3-13.8)	12.5 (11.2-13.8)	15.7 (1.7)	0.137
	65 and over	5.2 (4.5-6.1)	6.1 (5.2-7.0)	6.4 (5.6-7.2)	5.7 (5.0-6.4)	6.3 (5.6-7.1)	21.2 (1.1)	0.148
SES	Low/lowest quintile	8.6 (7.6-9.7)	9.7 (8.6-10.9)	10.2 (9.0-11.6)	10.2 (9.0-11.6)	10.1 (8.7-11.7)	17.4 (1.5)	0.080
	Middle quintile	9.9 (8.5-11.5)	13.1 (11.4-14.9)	11.1 (9.5-13.0)	12.8 (10.9-15.0)	13.5 (11.4-15.8)	36.4 (3.6)	0.020
	High/highest quintile	14.1 (10.6-12.0)	15.9 (14.6-17.3)	17.7 (16.1-19.3)	16.0 (14.6-17.4)	17.8 (16.1-19.5)	26.2 (3.7)	0.020
**Multiple risk factors (≥2/8)**								
*Overall*		62.4 (61.3-63.4)	61.4 (60.3-62.6)	59.3 (58.1-60.5)	60.3 (59.0-61.5)	59.8 (58.3-61.2)	-4.2 (-2.6)	<0.001
Sex	Males	64.3 (62.7-65.9)	64.5 (62.8-66.3)	61.3 (59.4-63.1)	62.3 (60.4-64.1)	63.4 (61.3-65.6)	-1.4 (-0.9)	0.062
	Females	60.4 (59.0-61.9)	58.3 (56.8-59.9)	57.3 (55.7-58.9)	58.3 (56.7-59.9)	56.2 (54.3-58.0)	-7.0 (-4.2)	<0.001
Age (y)	18 – 39	53.3 (51.2-55.5)	51.3 (48.9-53.7)	47.5 (44.9-50.1)	46.7 (44.2-49.3)	44.2 (41.0-47.4)	-17.1 (-9.1)	<0.001
	40 – 64	63.7 (62.2-65.2)	63.5 (61.9-65.0)	62.1 (60.6-63.6)	63.9 (62.2-65.6)	64.3 (62.5-66.2)	0.9 (0.6)	0.446
	65 and over	76.8 (75.3-78.3)	76.7 (75.2-78.2)	75.3 (73.8-76.7)	76.5 (75.2-77.8)	76.2 (74.9-77.5)	-0.8 (-0.6)	0.660
SES	Low/lowest quintile	68.3 (66.6-70.1)	67.7 (65.8-69.6)	66.0 (64.1-68.0)	68.2 (66.3-70.1)	66.1 (63.8-68.5)	-3.2 (-2.2)	0.107
	Middle quintile	63.1 (60.7-65.5)	62.5 (60.0-65.0)	63.2 (60.6-65.8)	61.0 (58.2-63.7)	60.8 (57.7-63.9)	-3.6 (-2.3)	0.062
	High/highest quintile	57.2 (55.5-58.9)	55.8 (53.9-57.6)	52.0 (50.1-53.9)	53.2 (51.3-55.0)	53.6 (51.4-55.8)	-6.3 (-3.6)	<0.001

*Respondents who had at least two of the following eight lifestyle risk factors: insufficient physical activity, long-term alcohol consumption, current smoking, under-consumption of fruits and vegetables, high blood pressure, high cholesterol, diabetes, and obesity.

All groups showed a decrease in those undertaking insufficient physical activity (Table 2) with the highest decrease for the younger age group (18–39 years). For inadequate nutrition the middle age group (40–64 years) showed a small increase in the proportion not consuming enough fruit and vegetables over the 10 years, while all other groups showed a decrease. The largest increase in the proportion consuming the recommended fruit and vegetables per day was for the 18- to 39-year-olds. There was a decrease in smoking prevalence in the 10 years with the greatest decrease being for females, 18- to 39-year-olds, and the high/highest SES quintile (most advantaged).

Only a small percentage of respondents qualify as having a long-term risk of harm from excessive alcohol although the relative change for 40- to 64-year-olds was 16.3% and that for 18- to 39-year-olds was −22.7% (Table 3). There were marked differences dependent upon the descriptive variable being assessed. The relative decrease of excessive soft drink consumption over the six years of data collection was substantial for all groups with later figures indicating over 10% of males, youngest age group, and the low/lowest SES quintile (most disadvantaged) reporting consuming over 500 ml of soft drink per day in 2012–2013. All groups showed an increase in the prevalence of obesity with the highest increase for the oldest age group (≥65 years) and the lowest increase for the middle SES quintile.

The proportion of adults reporting high blood pressure increased for all groups except the youngest age group with the highest relative change seen for males and the high/highest SES quintile (Table 4). High blood cholesterol readings were reported by over 17% of the participants with relative percentage changes increasing for all groups. The highest increase was for the low/lowest SES quintile. The unadjusted prevalence of diabetes over the 10 years increased for all groups. The highest relative change was for males and the youngest age group.

**Figure 1:**
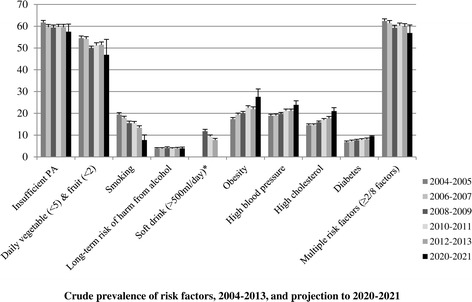
**Crude prevalence of risk factors, 2004–2013, and projection to 2020–2021.** *Data available from 2009 to 2013; insufficient data points for prediction.

**Figure 2:**
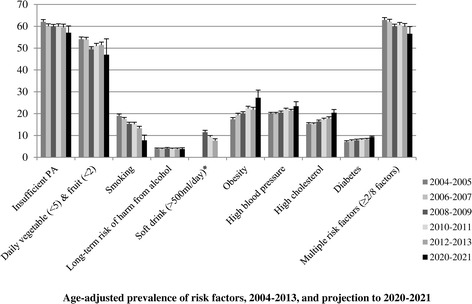
**Age-adjusted prevalence of risk factors, 2004–2013, and projection to 2020–2021.** *Data available from 2009 to 2013; insufficient data points for prediction.

The proportion having no risk factors increased for each group (Table 5). The youngest age group showed the biggest decrease in those reporting at least two of the risk factors. Figures 1 and 2 details the crude and age-adjusted overall prevalence for each risk factor over time and the 2020–2021 projections. While increases in physical activity and fruit and vegetable consumption and decreases in smoking prevalence and multiple risk factors are to be expected in 2020–2021, the prevalence of obesity, high blood pressure, high cholesterol, and diabetes are expected to increase.

## Discussion

This study describes the associations between major risk factors, overall and by age, sex, and a measure of SES. This analysis of major behavioral-related risk factors over 10 years has shown that overall adults in SA in 2012–2013 compared to 2004–2005 are more likely to be obese and to have high blood pressure, high cholesterol, or diabetes. They are also more likely to be undertaking physical activity, to be eating the required amounts of fruit and vegetables, and to be non-smokers. Overall alcohol long-term risk remained steady while the prevalence of multiple risk factors decreased. In addition, over the past six years of measurement, soft drink consumption of over 500 ml/day has also decreased. These associations remained stable when estimates were age-adjusted. Notable age group, sex, and SES differences were apparent.

Many of the results we found correspond with other literature although some major differences were apparent. We noted small significant increases in physical activity over the 10 year period for females, the two younger age groups, and the high SES category. Small increases in prevalence estimates were also reported in other studies with a major United States study also reporting no change for males and a small increase for females over 10 years using the National Health and Nutrition Examination Survey (NHANES) and the Behavioral Risk Factor Surveillance System (BRFSS) data [29]. A series of triennial physical activity-specific surveys undertaken in SA since 1998 showed similar trends [30]. As noted by the authors disparities between SES groups are of concern and warrant special attention. In a review of the impact of mass media campaigns on physical activity levels, Abioye et al. [10] reported increases in walking but no reduction in sedentary behavior, indicating the need for more targeted interventions.

Promoting awareness of the recommended number of servings of fruit and vegetables has formed the basis of major advertising campaigns conducted in SA and other states in Australia [31]. Complementing this are polices and state-wide programs to increase the supply of and demand for fruit and vegetables. Modest improvements in fruit and vegetable consumption were observed in our study. Ford et al. reported similar results in their assessment of fruit and vegetable intake over a 10-year period [9]. Of note was the positive relative change for the younger age group indicating their increase in reported fruit and vegetable consumption, perhaps based on the efforts of schools and campaigns targeted at younger age groups in the last decade. It should be noted that fruit and vegetable consumption is only a marker of good nutrition and does not adequately provide a comprehensive assessment of what should constitute an appropriate diet.

Overall smoking rates are declining in most developed countries [9,32,33] although concern is expressed for some demographic groups where rates are not necessarily falling [9]. Our results indicate some dramatic decreases with significant trends for each group analyzed. Self-reporting smoking estimates using the telephone as the mode of data collection often lack face validity when compared to face-to-face surveys. Surveillance systems such as SAMSS are not designed to provide concise and precise estimates in a population at a given point in time; for this, a census or large sample survey should be undertaken. For public health policy, the concern is mostly on increases and decreases of smoking behaviors over time. The reality in surveillance is that the population characteristics as well as the behaviors measured are changing over time, some more profoundly than others.

Some large differences were apparent for long-term risk of harm from adverse alcohol intake with promising significant decreases for the younger age group. The considerable efforts that have gone into alcohol health promotion efforts for this younger age group should be continued as a priority.

The increase in obesity over time has also been shown in many developed countries [7,9,29,32–34] although other studies have started to show a level of stabilization or even decreases [35,36]. Of interest in our data were the small decreases in the prevalence of obesity when comparing the latter two comparison periods (2012–2013 with 2010–2011), perhaps indicating a change in the direction of the trend. It should be noted that self-reported height and weight often do not correlate with actual measurements, as socially desirable responses are often given [37]. Notwithstanding, from a surveillance point of view, it is again the changes over time that are important to consider rather than the actual estimate, as socially desirable responses are usually consistently given.

In terms of self-reported ever-having high blood pressure, previous Canadian and Japanese studies have shown increases such as ours [32,33], although major biomedical studies highlighting global trends have shown decreases over the past three decades, [38] especially in high-income countries such as Australia. These decreases in high-income countries are due to the increased awareness of the consequences of high blood pressure, increased pharmacological treatment and diagnostic examinations, and the increase in the awareness and access to preventive services [39]. In interpreting our results, consideration that our study used the broad question of “have you ever been told you have high blood pressure” should be taken into account, while other clinical studies assess the high levels of medication interventions. Similarly, the increase in the prevalence of self-reported ever-having high cholesterol levels we report has been replicated in other studies, [32,33] although major global trends report decreases when current measurements are taken [40]. Again the differences are because of the questions we ask and the success of treatment regimes. It is also important to note that clarification on what respondents believe is “high cholesterol” may not be fully understood by respondents. Previous research has shown that the high cholesterol question used in SAMSS does not have very high validity or reliability values [41].

There was a potential bias from survey non-response in this study, and this should be seen as a weakness of the study. The sampling technique was unbiased other than the need for participants to be living in a household with a telephone, which now represents about 60% of Australian households [42]. There is a trend toward lower response rates in all types of population surveys as people protect their privacy or are overwhelmed by marketing telephone calls or mailings. The high number of mobile-only households not necessarily included in this sample could lead to bias, as mobile-only households are associated with younger populations who often have higher risk factor rates, especially smoking and excessive alcohol [42]. Additional file 1 details the unweighted age and sex profile over the years highlighting the increased proportion of the younger age group not responding to this survey. This bias needs to be taken into account when assessing the results of this research. In addition, the cross-sectional nature of the data collection limits interpretation of the results to associations only. The self-reported nature of the data collection is also acknowledged as a weakness of the study with the known subtleties associated with persons over- or under-reporting their behaviors [37]. A further limitation of the study includes the inability to include not only risk factors but other data available within SAMSS, including chronic conditions, general health, health services, and mental and social health indicators. It should also be noted as a weakness that SAMSS is not collecting data on some of the other important risk factors including salt consumption, [3] as well as duration and intensity of each risk factor. In interpreting the results it should also be noted that the population has aged over this period, and population-based interventions take time to show positive results.

The strengths of this study are the large sample size, the use of standardized validated instruments that have not altered over the period of data collection, and the stability of the methodology over the research period. The study also highlights the advantages of ongoing continuous surveillance system with limited setup and management costs. Future analysis of these data would benefit from assessing trends over time with mortality figures.

## Conclusion

This study describes the associations between major risk factors overall and by age, sex, and measures of SES. While individual population-wide self-report studies on specific risk factor trends have been reported [7,14] or multiple risk factor comparisons on specific populations [43,44], comparisons over time on a range of risk factors of the whole population are less common. This study used 10 years of continuous data collection to highlight changes in trends in risk factor prevalence. It would seem that public health efforts in increasing the proportion of the population undertaking appropriate risk factor behavior is succeeding, with trends from 2004 to 2013 showing encouraging trends. Notwithstanding, there are some “big questions” to address. The “fight” against risk factors seems to have produced some results in the right direction over the last decade but still some indicators of population health outcomes are getting worse. To answer this potential contradiction is out of the scope of this paper. Nevertheless, hypotheses can be advanced, including that the magnitude of the positive changes is, as yet, not sufficient to produce significant changes, and the influence of contextual unmeasured variables (urban sprawl, increased sedentary activity such as computer games). Deriving comparable trends over time and by key demographics and SES variables provides evidence for policymakers and health planners to encourage interventions aimed at preventing chronic disease.

## Additional file

Additional file 1:
**Demographic characteristics of respondents aged 18 years and over, 2004 to 2013, weighted and unweighted.**

## Abbreviations

BRFSS: Behavioral Risk Factor Surveillance System
CATI: Computer-Assisted Telephone Interviewing
CVD: Cardiovascular Disease
NCD: Non-Communicable Disease
NHANES: National Health and Nutrition Examination Survey
SA: South Australia
SAMSS: South Australian Monitoring and Surveillance System
SEIFA: Social Economic Index for Areas
SES: Socio-Economic Status
SPSS: Statistical Package for Social Sciences
