# Supplementary material for: Ten-year trends in major lifestyle risk factors using an ongoing population surveillance system in Australia
Source: Popul Health Metr. 2014 Oct 30;12:31. doi: 10.1186/s12963-014-0031-z (PMC4222399; doi:10.1186/s12963-014-0031-z)
Supplement: Additional file 1: — Demographic characteristics of respondents aged 18 years and over, 2004 to 2013, weighted and unweighted. [file 12963_2014_31_MOESM1_ESM.docx]

[**Appendix 1: Demographic characteristics of respondents aged 18 years and over, 2004 to 2013**](http://www.nhmrc.gov.au)**, weighted and unweighted**

[**1: Age and Sex profile of respondents aged 18 years and over, 2004 to 2013**](http://www.nhmrc.gov.au) **- weighted**

|  | [**Year**](http://www.nhmrc.gov.au) | | | | |
| --- | --- | --- | --- | --- | --- |
|  | [**2004-2005**](http://www.nhmrc.gov.au) | [**2006-2007**](http://www.nhmrc.gov.au) | [**2008-2009**](http://www.nhmrc.gov.au) | [**2010-2011**](http://www.nhmrc.gov.au) | [**2012-2013**](http://www.nhmrc.gov.au) |
| [**Age group** (%)](http://www.nhmrc.gov.au) |  |  |  |  |  |
| 18 – 39 | 37.3 | 36.9 | 36.4 | 35.4 | 34.2 |
| 40 – 64 | 43.2 | 43.3 | 43.7 | 44.6 | 45.0 |
| 65 and over | 19.5 | 19.7 | 19.9 | 20.1 | 20.8 |
| [**Sex** (%)](http://www.nhmrc.gov.au) |  |  |  |  |  |
| [Males](http://www.nhmrc.gov.au) | [48.8](http://www.nhmrc.gov.au) | [49.1](http://www.nhmrc.gov.au) | [48.8](http://www.nhmrc.gov.au) | [48.7](http://www.nhmrc.gov.au) | [48.6](http://www.nhmrc.gov.au) |
| [Females](http://www.nhmrc.gov.au) | [51.2](http://www.nhmrc.gov.au) | [50.9](http://www.nhmrc.gov.au) | [51.2](http://www.nhmrc.gov.au) | [51.3](http://www.nhmrc.gov.au) | [51.4](http://www.nhmrc.gov.au) |

**[2: Age and Sex profile of respondents aged 18 years and over, 2004 to 2013](http://www.nhmrc.gov.au), unweighted**

|  | [**Year**](http://www.nhmrc.gov.au) | | | | |
| --- | --- | --- | --- | --- | --- |
|  | [**2004-2005**](http://www.nhmrc.gov.au) | [**2006-2007**](http://www.nhmrc.gov.au) | [**2008-2009**](http://www.nhmrc.gov.au) | [**2010-2011**](http://www.nhmrc.gov.au) | [**2012-2013**](http://www.nhmrc.gov.au) |
| [**Age group** (%)](http://www.nhmrc.gov.au) |  |  |  |  |  |
| 18 – 39 | 23.1 | 19.7 | 16.2 | 16.8 | 13.3 |
| 40 – 64 | 46.7 | 47.9 | 48.7 | 44.2 | 42.3 |
| 65 and over | 30.2 | 32.4 | 35.1 | 39.0 | 44.4 |
| [**Sex** (%)](http://www.nhmrc.gov.au) |  |  |  |  |  |
| [Males](http://www.nhmrc.gov.au) | 44.2 | 42.8 | 42.6 | 42.6 | 40.6 |
| [Females](http://www.nhmrc.gov.au) | 55.8 | 57.2 | 57.4 | 57.4 | 59.4 |
